# Metabolomics analysis of post-traumatic stress disorder symptoms in World Trade Center responders

**DOI:** 10.1038/s41398-022-01940-y

**Published:** 2022-04-28

**Authors:** Pei-Fen Kuan, Xiaohua Yang, Roman Kotov, Sean Clouston, Evelyn Bromet, Benjamin J. Luft

**Affiliations:** 1 Department of Applied Mathematics and Statistics, Stony Brook University, Stony Brook, NY USA; 2 Department of Medicine, Stony Brook University, Stony Brook, NY USA; 3 Department of Psychiatry, Stony Book University, Stony Brook, NY USA; 4 Department of Family, Population and Preventive Medicine, Stony Book University, Stony Brook, NY USA

**Keywords:** Genomics, Diagnostic markers

## Abstract

Metabolomics has yielded promising insights into the pathophysiology of post-traumatic stress disorder (PTSD). The current study expands understanding of the systems-level effects of metabolites by using global metabolomics and complex lipid profiling in plasma samples from 124 World Trade Center responders (56 PTSD, 68 control) on 1628 metabolites. Differential metabolomics analysis identified hexosylceramide HCER(26:1) associated with PTSD at FDR < 0.1. The multi-metabolite composite score achieved an AUC of 0.839 for PTSD versus unaffected control classification. Independent component analysis identified three metabolomic modules significantly associated with PTSD. These modules were significantly enriched in bile acid metabolism, fatty acid metabolism and pregnenolone steroids, which are involved in innate immunity, inflammatory process and neuronal excitability, respectively. Integrative analysis of metabolomics and our prior proteomics datasets on subsample of 96 responders identified seven proteomic modules significantly correlated with metabolic modules. Overall, our findings shed light on the molecular alterations and identify metabolomic-proteomic signatures associated with PTSD by using machine learning and network approaches to enhance understanding of the pathways implicated in PTSD. If present results are confirmed in follow-up studies, they may inform development of novel treatments.

## Introduction

Post-traumatic stress disorder (PTSD) is a debilitating condition that can occur after exposure to a traumatic event. The estimated lifetime prevalence of PTSD among adult Americans is 6.1% [1]. Many people with PTSD do not respond to existing treatments [2, 3], as highlighted by the virtually unchanged prevalence rates of PTSD among World Trade Center (WTC) responders over the past two decades [4, 5], thus placing them at risk of chronic disability and long-term cognitive, social and occupational impairments [6–9]. As such, there is a critical need to understand biological processes that maintain PTSD in order to identify novel biomarkers to aid illness monitoring, improve evaluation of response to treatment, and provide targets for treatment development. Our group had previously found that PTSD is associated with serological proteinopathy in a proteomics study consisting of 276 plasma proteins with known involvement in neurobiological processes, immunology, cardiovascular, inflammatory and metabolism [10].

Metabolomics has emerged as a promising alternative strategy for biomarker discovery [11–14]. Unlike transcriptomics or proteomics, which are subjected to further epigenetic or posttranslational modifications, the metabolome represents the final outcomes of gene expression and serves as a direct signature of biochemical activity; therefore, its readout is close to the phenotype and is highly informative regarding an individual’s overall health status [15, 16]. PTSD and physical illnesses, particularly metabolic syndrome [17, 18], are frequently comorbid. Thus, the metabolomics profiles of people with PTSD may differ from those of healthy individuals.

Despite the advantages of metabolomics, metabolomic profiling in patients with PTSD remains scarce. To our knowledge, only three such studies have been performed in humans to date. The first was conducted with 20 patients with PTSD and 18 health controls matched by age and ethnicity; 60 metabolites were analyzed, and 13 differential metabolites were identified at nominal *p* < 0.05 [19]. The second study was conducted with male US combat veterans, 83 with PTSD and 82 non-PTSD controls; 244 metabolites were examined. The authors identified metabolites involved in glycolysis, fatty acid uptake and metabolism, and suggested that mitochondrial dysfunctions were a key contributor to the differences between PTSD and controls [20]. The third study was conducted with 102 male Croatian combat veterans with PTSD and 102 healthy controls; two metabolites were significantly higher in the PTSD group than the control group [21]. However, there was no overlap in the metabolites identified by these three studies. Additionally, the first study found that the multi-metabolite composite score based on 19 metabolites achieved 85% accuracy in PTSD classification [19], but the classification model was trained on a very small sample size (20 PTSD, 18 controls) and was evaluated on the same training sample rather than an independent replication sample. Thus, the reported classification accuracy was potentially overestimated, and the performance of a multi-metabolite composite score remains unknown.

The purpose of the current study was to extend the scope of existing studies by investigating the associations of metabolites with PTSD and expanding the metabolomics and lipidomics coverage through an unbiased profiling approach, constructing a multi-metabolite composite score, and evaluating model performance in an unbiased manner. To this end, we analyzed plasma samples from WTC responders (56 PTSD, 68 controls) who were exposed to the same disaster, the 9/11 attacks in New York City, thus minimizing the heterogeneity of the trauma. We also report the first integration of metabolomics with proteomics using network analysis to better understand the pathways implicated by PTSD.

## Methods

### Participants and PTSD assessment

Participants were recruited through the Stony Brook WTC-Health Program [22]. The current study was approved by the Stony Brook University IRB, and written informed consent was obtained from all participants. Blood samples were obtained during 2018–2019, 17–18 years after the WTC collapse. The inclusion criteria were adequate English language skills to complete the protocol and being male. We included only men because women show notably different metabolomic patterns [23], and <10% of responders in the Stony Brook cohort were women. Exclusion criteria were diagnosis of cognitive impairment and head injuries.

Probable PTSD was measured with the Posttraumatic Stress Disorder Checklist-Specific Version (PCL-17) [24], a 17-item self-reported questionnaire modified to assess the severity of WTC-related DSM-IV PTSD symptoms over the past month on a scale of 1 (never bothered by) to 5 (extremely bothered by) (Cronbach α = 0.96). Probable PTSD was operationalized with a PCL total score >44. The unaffected sample was asymptomatic (PCL total score <22) and was subject to an additional medical record review to rule out responders with a clinical history of related psychiatric disorders. A total of 124 participants were included (56 with PTSD and 68 trauma-exposed controls). All of the participants were non-smokers, 87% were Caucasian, and the mean age was 54.5 years (SD = 7.8). Medical conditions and dust cloud exposure [25] were also reported for these participants. The 124 samples were divided into two subsamples. The first subsample (discovery) consisted of 39 participants with PTSD and 49 trauma-exposed controls, and the second subsample (replication) consisted of 17 participants with PTSD and 19 trauma-exposed controls.

### Blood sampling

Whole blood samples were collected into anticoagulant K2-EDTA blood collection tubes (BD Vacutainer, Franklin Lakes, NJ) and inverted eight to ten times, then centrifuged at 2000 × *g* for 15 min at 4 °C. Plasma samples were then separated and placed in polyethylene tubes stored at −80 °C.

### Metabolomics profiling

Metabolomics in the plasma samples was profiled with two Metabolon panels; the discovery HD4 metabolomics panel (HD4) and the complex lipid panel (CLP) (https://www.metabolon.com). Additional details on the Metabolon platform including sample preparation, data extraction and compound identification are described in Evans, DeHaven [26, 27]. The data were normalized to the volume extracted, log transformed and imputed with Metabolon mView software for each batch separately. A total of 738 and 890 metabolites of known identity from the HD4 and CLP panel, respectively, present in both subsamples, were included in subsequent analysis.

### Differential metabolomics analysis

Linear regression was fitted for each metabolite as the dependent variable, PTSD status as the independent variable, with adjustment for age, race and (objective) body mass index (BMI) in the discovery and replication subsamples separately. All continuous variables were standardized. Statistically significant metabolites were identified at FDR < 0.1 in the discovery subsample and were considered to be replicated if *p* < 0.05 in the replication subsample. The analysis was repeated by combining the discovery and replication subsamples, and subsample effects were removed with the ComBat program [28]. We further compared our results to the metabolites reported in the three prior metabolomics studies [19–21].

Among the identified metabolites, sensitivity analysis was conducted to evaluate the association after additional adjustments for each medical condition, as well as dust cloud exposure [25].

### Multi-metabolite composite score

To evaluate the utility of metabolomics in classifying PTSD versus control, we applied the elastic net algorithm [29]. The discovery subsample was used as the training set, and the replication subsample was used as the test set. Within the training set, *t*-tests were used to rank the metabolites. The top K metabolites from CLP and HD4 panels ranked by *p*-values from t-tests were used as candidate feature sets. The optimal tuning parameters were determined via fivefold cross-validation in the training set. The resulting model was used to predict the score in the test set. The area under the ROC curve (AUC) was used as a metric for performance evaluation. We varied *K* = 2, 3, …, 10, 20, 30, …, 90, 100, 200, 300, 400 and 500, selected from each panel as well as combined CLP + HD4 panels. We also ran the elastic net models by including all metabolites in CLP, HD4 and CLP + HD4 panels. For the optimal model (i.e., the model with the largest AUC in the test set), we fitted a regression model using the multi-metabolite composite score as the dependent variable and PTSD status as the independent variable, adjusting for age, race and BMI.

### Integration with proteomics

Among the 124 participants, 96 (30 PTSD, 66 controls) had matching proteomics data from our previous study [10]. Briefly, the proteomics profiling was conducted with the Olink Proseek Multiplex Platform. A total of 276 proteins were targeted, involving a range of processes indicative of neurological diseases, cellular regulation, immunology, cardiovascular, inflammatory, development and metabolism functions. Additional details on the proteomics dataset can be found in our previous study [10]. To understand the molecular interactions between proteomics and metabolomics, we first performed independent component analysis (ICA) on subsample adjusted metabolomics to identify modules of metabolite co-expression, and then repeated same analyses for proteins. ICA has been shown to be a powerful approach compared to several alternatives (including the weighted gene co-expression analysis (WGCNA) [30] and biclustering method) in module detection [31]. We used the fastICA algorithm [32] with 50 runs to ensure the robustness of the independent components (ICs) identified, i.e, only ICs replicated across runs were retained. The number of components were estimated to account for 80% of the variance. Important metabolites contributing to each component were defined as the metabolites with estimated source signal greater than 2 standard deviations [33]. For each identified module from the metabolomics data, we fitted a regression model by using the estimated source signal as the dependent variable and PTSD status as the independent variable, adjusting for age, race and BMI. Statistically significant metabolomic modules associated with PTSD were identified at *p* < 0.05. Next, Pearson correlation coefficients were computed between metabolomic module IC to proteomic module IC. Statistically significant associations were identified at *p* < 0.05. To further understand the functional mechanisms of metabolites, we performed over-representation analyses via hypergeometric tests to identify the metabolic pathways associated with the metabolites in each module. Statistically significant pathways were identified at *p* < 0.05.

## Results

### Participant characteristics

The participants with PTSD were 3 years older than the trauma-exposed control participants (*p* = 0.028). No significant differences in race or BMI were observed between the PTSD and trauma-exposed control groups (Table 1).

**Table 1:** Clinical characteristics of samples in discovery and replication subsamples. The *p*-values were computed from t-tests (for age and BMI) and chi-squared test (for race).

All	PTSD *N* = 56	Control *N* = 68	*P*-value
Age			
Mean (SD)	56.2 (8.0)	53.1 (7.4)	0.028
Race *N* (%)			
Caucasian	51 (91.1)	57 (83.8)	0.353
Other	5 (8.9)	11 (16.2)	
BMI			
Mean (SD)	31.6 (4.8)	30.8 (4.7)	0.319
**Discovery**	**PTSD** ***N*** = 39	**Control** ***N*** = 49	***P*-value**
Age			
Mean (SD)	55.7 (7.8)	52.7 (7.6)	0.071
Race *N* (%)			
Caucasian	35 (89.7)	41 (83.7)	0.609
Other	4 (10.3)	8 (16.3)	
BMI			
Mean (SD)	31.2 (4.8)	31.0 (4.6)	0.841
**Replication**	**PTSD** ***N*** = 17	**Control** ***N*** = 19	***P*-value**
Age			
Mean (SD)	57.4 (8.4)	54.3 (7.1)	0.238
Race *N* (%)			
Caucasian	16 (94.1)	16 (84.2)	0.680
Other	1 (5.9)	3 (15.8)	
BMI			
Mean (SD)	32.5 (5.1)	30.1 (5.0)	0.153

Additionally, no significant differences in dust cloud exposure, incidence of gastroesophageal reflux disease (GERD), diabetes, heart disease, systolic and diastolic blood pressures, as well as levels of HDL, LDL, VLDL, triglycerides, total cholesterol and creatinine between the PTSD and trauma-exposed control groups (Supplementary Table 1). Participants with PTSD had higher rate of hypertension than trauma-exposed controls (Supplementary Table 1).

### Differentially expressed metabolites associated with PTSD

Hexosylceramide (HCER (26:1)) was identified at FDR < 0.1 in the discovery subsample. This metabolite had a consistent effect size direction in the replication subsample although the *p*-value did not reach statistical significance. In the combined analysis, HCER(26:1) was also significant at FDR < 0.1 (Table 2). Sensitivity analysis showed that HCER(26:1) remained significant (*p* < 0.001 in the combined analysis) after additional adjustments for each medical condition and dust cloud exposure (Supplementary Table 2). BMI was not associated with HCER(26:1) (*p* = 0.836 in the combined analysis).

**Table 2:** List of metabolites retained in differential metabolomics analysis (HCER (26:1)) or multi-metabolite composite score (5-oxoproline, 6-oxopiperidine-2-carboxylate, beta-hydroxyisovalerate, caproate (6:0) and glycocholate).

			Discovery			Replication		Combined		
Metabolite	Super pathway	Sub pathway	Coef	*p*	FDR	Coef	*p*	Coef	*p*	FDR
HCER (26:1)	Sphingolipids	Hexosylceramide	0.86	<0.001	0.03	0.47	0.21	0.74	<0.001	0.06
5-oxoproline	Amino Acid	Glutathione Metabolism	0.67	0.001	0.21	0.62	0.09	0.65	<0.001	0.13
6-oxopiperidine-2-carboxylate	Amino Acid	Lysine Metabolism	0.62	0.003	0.21	0.69	0.06	0.62	<0.001	0.15
beta-hydroxyisovalerate	Amino Acid	Leucine, Isoleucine and Valine Metabolism	0.76	<0.001	0.18	0.43	0.24	0.63	<0.001	0.15
caproate (6:0)	Lipid	Medium Chain Fatty Acid	0.72	<0.001	0.19	0.26	0.5	0.58	0.001	0.25
glycocholate	Lipid	Primary Bile Acid Metabolism	0.66	0.002	0.21	0.71	0.05	0.66	<0.001	0.13

Four of the 13 metabolites that were reported in the PTSD study of Karabatsiakis, Hamuni [19] were analyzed in our study. The association of Glycocholate was *p* < 0.05 in our study. However, the effect size was opposite from that reported in Karabatsiakis, Hamuni [19]. Nine metabolites were associated with PTSD in the study by Mellon et al. [20] and none was significantly associated with PTSD in our study. Two glycerophospholipids (PE(18:1/0:0) and PC(18:1/0:0)) were associated with PTSD in the study of Konjevod et al. [21]; however, these two metabolites were not detected or retained in the CLP panel and therefore were not included in our analysis (Supplementary Table 3).

Among the 1628 metabolites analyzed in our study, 61 were sphingolipids. Only HCER (26:1) was significant at FDR < 0.1. 57 out of 61 sphingolipids showed higher expression in PTSD (Supplementary Table 4). Results remained consistent with >55 sphingolipids showing higher expression in PTSD after additional adjustments for GERD, heart disease, hypertension, creatinine and dust cloud exposure, whereas adjustments for other medical conditions yield >40 sphingolipids showing higher expression in PTSD (Supplementary Table 4). On the other hand, 9 sphingolipids were significantly associated with BMI at FDR < 0.1 (Supplementary Table 4).

### Multi-metabolite composite score

**Fig. 1:**
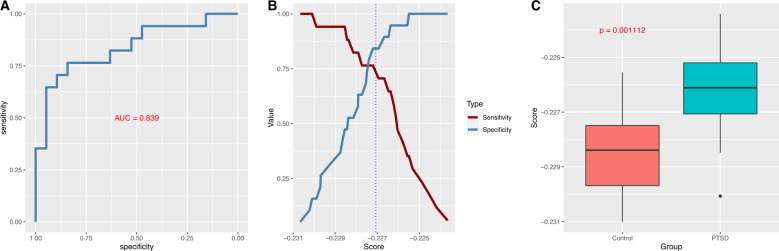
Multi-metabolite model evaluation. **A** AUC plot in the test set. **B** Plot of sensitivity versus specificity. The vertical dotted line corresponds to the optimal cutoff according to the Youden method. **C** Boxplot comparing the multi-metabolite composite score in the test set.

Across different candidate feature sets (i.e., the number of top K metabolites included in the elastic net model), the model constructed using the top 5 metabolites from the HD4 panel as candidate features yielded the largest AUC, namely, 0.839 in the test set (Fig. 1A). The optimal Youden index J for the elastic net prediction model was 0.607 (sensitivity = 0.767, specificity = 0.842, positive predictive value = 0.813 and negative predictive value = 0.800) (Fig. 1B). These 5 metabolites were 5-oxoproline, 6-oxopiperidine-2-carboxylate, beta-hydroxyisovalerate, caproate (6:0) and glycocholate (Table 2), and were not associated with BMI (*p* > 0.05 in the combined analysis). The effect sizes of these 5 metabolites remained consistent after additional adjustments for medical conditions and dust cloud exposure (Supplementary Table 2). The multi-metabolite composite score was also significantly associated with PTSD status in the test set (coef = 1.07, *p* = 0.001, Fig. 1C). In comparison, at the individual metabolite level, HCER(26:1) had the largest AUC, 0.734, in the training data from the CLP panel; however, the AUC in the test data was only 0.598. In contrast, beta-hydroxyisovalerate had the largest AUC (0.722) in the training data from the HD4 panel; however, the AUC in the test data was somewhat lower (0.669).

### Integrative proteomics-metabolomics analysis

**Fig. 2:**
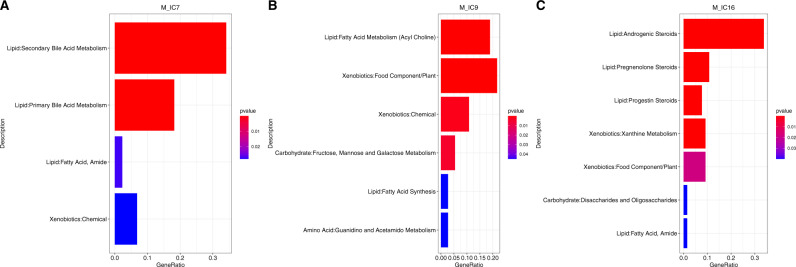
Metabolic pathway analyses. In each plot, the length of the bar corresponds to the ratio of the number of metabolites in the pathway to the number of metabolites in the module, whereas the color corresponds to the p-value gradient. The bars are ordered by *p*-values. **A** Bar plot showing the list of significant pathways for the M_IC7 module. **B** Bar plot showing the list of significant pathways for the M_IC9 module. **C** Bar plot showing the list of significant pathways for the M_IC16 module.

The ICA algorithm identified 16 modules of co-expressed metabolites and 19 modules of co-expressed proteins. Among the 16 modules of co-expressed metabolites, 3 modules (M_IC7, M_IC9 and M_IC16) were significantly associated with PTSD status at *p* < 0.05. M_IC7, M_IC9 and M_IC16 modules consisted of 44, 37 and 65 metabolites, respectively (Supplementary Table 5). M_IC7 was enriched in the bile acid metabolism, M_IC9 was enriched fatty acid metabolism and synthesis, and M_IC16 was enriched in several steroids (Fig. 2).

**Fig. 3:**
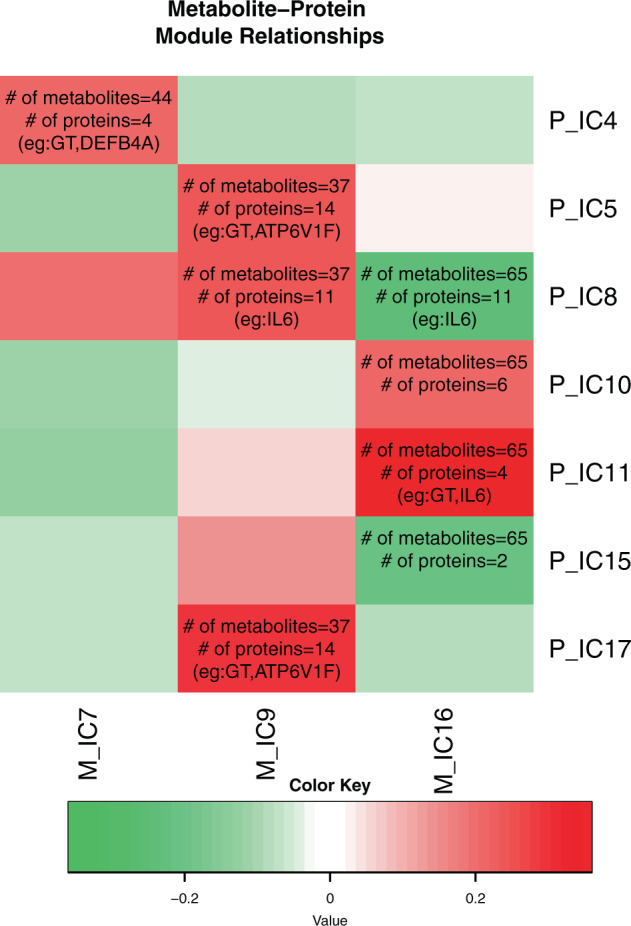
Heatmap depicting the correlations among the 3 metabolomic modules significantly associated with PTSD status and 7 proteomic modules which are correlated with these 3 metabolomic modules. The correlations with *p* < 0.05 corresponded to the cell with texts.

One proteomic module (P_IC4) was significantly correlated with metabolomic module M_IC7, three proteomic modules (P_IC5, P_IC8 and P_IC17) were significantly correlated with metabolomic module M_IC9, and four protein modules (P_IC8, P_IC10, P_IC11 and P_IC15) were significantly correlated with metabolomic module M_IC16; yielding a total of 7 unique proteomic modules (Fig. 3). Among the proteins in these proteomic modules, DEFB4A (P_IC4) and ATP6V1F (P_IC5, P_IC17) were associated with PTSD in our previous study [10]. Other proteins which emerged from these 7 proteomic modules included gastrotropin (GT) and IL6 which were identified in 4 (i.e., P_IC4, P_IC5, P_IC11, P_IC17) and 2 (i.e., P_IC8, P_IC11) modules, respectively. The correlation heatmap of all pairwise metabolomic and proteomic modules is provided in Supplementary Fig. 1. The list of proteins and metabolites, along with the estimated source signal in each module are provided in Supplementary Table 5.

## Discussion

Growing evidence links PTSD to a host of systemic illnesses including metabolic syndrome [17, 18], thus suggesting that dysregulated metabolites may be detected in people with PTSD. The current study used an unbiased approach to profile a large set of metabolites and complex lipids. We found HCER(26:1), a hexosylceramide which belongs to the class of sphingolipids associated with PTSD. Hexosylceramide has been found to be associated with mild traumatic brain injury and PTSD, and the effects are influenced by APOE e4 status [34]. Sphingolipids are an important component of the plasma membrane, are highly enriched in the central nervous system (CNS), play a key role in maintaining neuronal health [35] and mediate the inflammatory process [36]. Plasma sphingolipids have been found to be overexpressed in people with PTSD compared with control individuals [37]. Although HCER(26:1) was the only sphingolipid meeting the FDR threshold in this study, 57 of the 61 sphingolipids analyzed showed higher expression in PTSD, thus suggesting that the link between inflammation and PTSD may be mediated by sphingolipids. The current study did not replicate the findings of metabolites significantly associated with PTSD reported in previous studies [19–21], possibly in part because of sample differences in sex, age, race/ethnicity and time since trauma, populations sampled, PTSD measure used, and differences in the clinical profiles of participants (PTSD is a heterogeneous disorder).

The multi-metabolites composite score based on 5 metabolites (5-oxoproline, 6-oxopiperidine-2-carboxylate, beta-hydroxyisovalerate, caproate (6:0) and glycocholate) achieved an AUC of 0.839 in PTSD classification, which was higher compared to the AUCs obtained from PTSD classification by either gene expression or proteomics in our previous studies [10, 38]. The current multi-metabolites composite score had relatively high sensitivity and specificity, and thus might serve as an informative screening and diagnosis tool. Although promising, the multi-metabolites composite needs to be tested in other populations with PTSD before its clinical utility can be verified.

All five metabolites included in the multi-metabolites composite were upregulated in PTSD. Beta-hydroxyisovalerate is an amino acid in the leucine, isoleucine and valine metabolism pathway and is associated with biotinidase deficiency [39]. Leucine, isoleucine and valine have been implicated in chronological lifespan [40], whereas biotinidase deficiency is associated with neurological symptoms, and developmental and behavioral issues, including stress and anxiety [41] and autism [42]. On the other hand, 5-oxoproline, also known as pyroglutamic acid, is an amino acid involved in glutathione metabolism. Glutathione metabolism plays a part in several mechanisms including antioxidant defense, nutrient metabolism, and the regulation of cellular events, such as cytokine production and immune response; moreover, glutathione deficiency is linked to oxidative stress, an important mechanism associated with aging and the pathogenesis of different diseases [43]. Additionally, glutathione concentration has been found to be higher in both the prefrontal cortex and anterior cingulate cortex in people with PTSD [44]. 6-oxopiperidine-2-carboxylate is an amino acid involved in lysine metabolism; caproate (6:0) is a fatty acid; whereas glycocholate is involved in primary bile acid metabolism.

The ICA analysis identified three modules of co-expressed metabolites which were significantly associated with PTSD. These modules were enriched in the bile acid metabolism, fatty acid metabolism and synthesis, and pregnenolone steroids, respectively. Fatty acid metabolism is involved in both pro- and anti-inflammatory processes [45, 46], whereas bile acid metabolism is involved in innate immunity and inflammation [47–49], suggesting that these metabolic pathways may contribute to PTSD via dysregulated neuroinflammation. On the other hand, pregnenolone steroid functions is a metabolic intermediate in the biosynthesis of several the steroid hormones [50], notably glucocorticoids, an important compound associated with PTSD [51]. Pregnenolone is also a neurosteroid which modulates neuronal excitability and has emerged as a promising therapeutic target in schizophrenia [52].

The three metabolomic modules were correlated with one, three and four proteomic modules, respectively, yielding a total of 7 unique proteomic modules. Among these 7 protein modules, several interesting proteins emerged, including gastrotropin, IL6 and ATP6V1F which were identified in 4, 2 and 2 proteomic modules, respectively. Gastrotropin is a fatty and bile acid binding protein, which partially explains the associations between these 4 proteomic modules and the three metabolomic modules; since metabolic pathway analyses also identified fatty acid and bile acid metabolism pathways to be enriched in these metabolomic modules. On the other hand, IL6 is a proinflammatory cytokine which is implicated in PTSD [53, 54], whereas ATP6V1F is involved in regulation of luminal or extracellular acidification, a crucial process for the normal physiological function of several organs [55] and is implicated PTSD [56, 57], including in our previous study [10].

### Strengths and limitations

The current study has several strengths, including an unbiased profiling of metabolomics and lipidomics through validated Metabolon HD4 and CLP panels, unbiased construction and evaluation of multi-metabolite composite scores, the integration of metabolomics and proteomics, and a common trauma across PTSD cases and controls. Nonetheless, our findings must be considered in the context of several limitations. First, our study was conducted with a cross-sectional design; therefore, we cannot determine whether the observed associations with current PTSD, defined by the presence of DSM-IV-related symptom severity, were a consequence of the disorder or a part of its etiology. Comparisons to trauma-exposed controls suggest that differential metabolomics is not just a consequence of trauma, but a longitudinal design is needed to determine the direction of the association with PTSD. Second, the participants were male responders to the WTC disaster. Although this aspect improves the biological homogeneity of analyses, it constrains the potential generalizability of our results to other traumatized groups, and to women. Larger and more diverse cohorts with other trauma experiences across both sexes are needed to determine the effects of sex and to replicate the findings of differential metabolomics analysis associated with PTSD. Lastly, future pre-post studies are needed to evaluate whether the constructed multi-metabolites composite score can predict onset and/or chronicity of PTSD after trauma exposure.

## Conclusions

The current study expanded metabolomics and lipidomics coverage by using an unbiased profiling approach to identify metabolites associated with PTSD. Our study further derived a multi-metabolite composite score that was evaluated in an unbiased manner in a replication subsample. The integrative analysis of proteomics and metabolomics complemented the univariate biomarker analysis and provided insights from a system biology perspective by characterizing the molecular interactions between the multi-omics datasets. Replication studies are needed to test the robustness of the composite score which, if confirmed, may aid in PTSD screening, diagnosis, and monitoring.

## Supplementary information

Supplementary Table 1

Supplementary Table 2

Supplementary Table 3

Supplementary Table 4

Supplementary Materials

Supplementary Figure 1

Supplementary Table 5

## Data Availability

The metabolomics data is available at Synapse (https://www.synapse.org/#!Synapse:syn26532705, doi:10.7303/syn26532705).
