# Supplementary material for: Metabolomics analysis of post-traumatic stress disorder symptoms in World Trade Center responders
Source: Transl Psychiatry. 2022 Apr 28;12:174. doi: 10.1038/s41398-022-01940-y (PMC9050707; doi:10.1038/s41398-022-01940-y)
Supplement: Supplementary file 5 — Supplementary Materials [file 41398_2022_1940_MOESM5_ESM.docx]

**Supplementary Figure Legend**

**Supplementary Figure 1.** Heatmap depicting the correlations among the 16 metabolomic modules and 19 proteomic modules. The correlations with p < 0.05 were marked with *.

**Supplementary Table Legends**

**Supplementary Table 1.** Clinical characteristics of samples in discovery and replication subsamples with respect to medical conditions and dust cloud exposure. The p-values were computed from t-tests (for systolic BP, diastolic BP, HDL, LDL, VLDL, triglycerides, total cholesterol and creatinine) and chi-squared tests (for dust cloud, GERD, hypertension, diabetes and heart disease).

**Supplementary Table 2**. List of metabolites retained in differential metabolomics analysis (HCER (26:1)) or multi-metabolite composite score (5-oxoproline, 6-oxopiperidine-2-carboxylate, beta-hydroxyisovalerate, caproate (6:0) and glycocholate) from the combined analysis with additional adjustments for each medical condition and dust cloud exposure.

**Supplementary Table 3.** Comparison of results to those from published PTSD metabolomics studies ^19-21^. NA indicates that the metabolite was not found in our study.

**Supplementary Table 4.** List of sphingolipids along with the estimated coefficients, p-values and FDR associated with PTSD and BMI from differential metabolomics analysis, as well as the results from analyses with additional adjustments for each medical condition and dust cloud exposure.

**Supplementary Table 5.** List of proteins and metabolites, along with the estimated source signal in each module from the ICA analysis.
