# Supplementary figures and images for: Metabolomics analysis of post-traumatic stress disorder symptoms in World Trade Center responders
Source: Transl Psychiatry. 2022 Apr 28;12:174. doi: 10.1038/s41398-022-01940-y (PMC9050707; doi:10.1038/s41398-022-01940-y)

# Metabolite-Protein Module Relationships

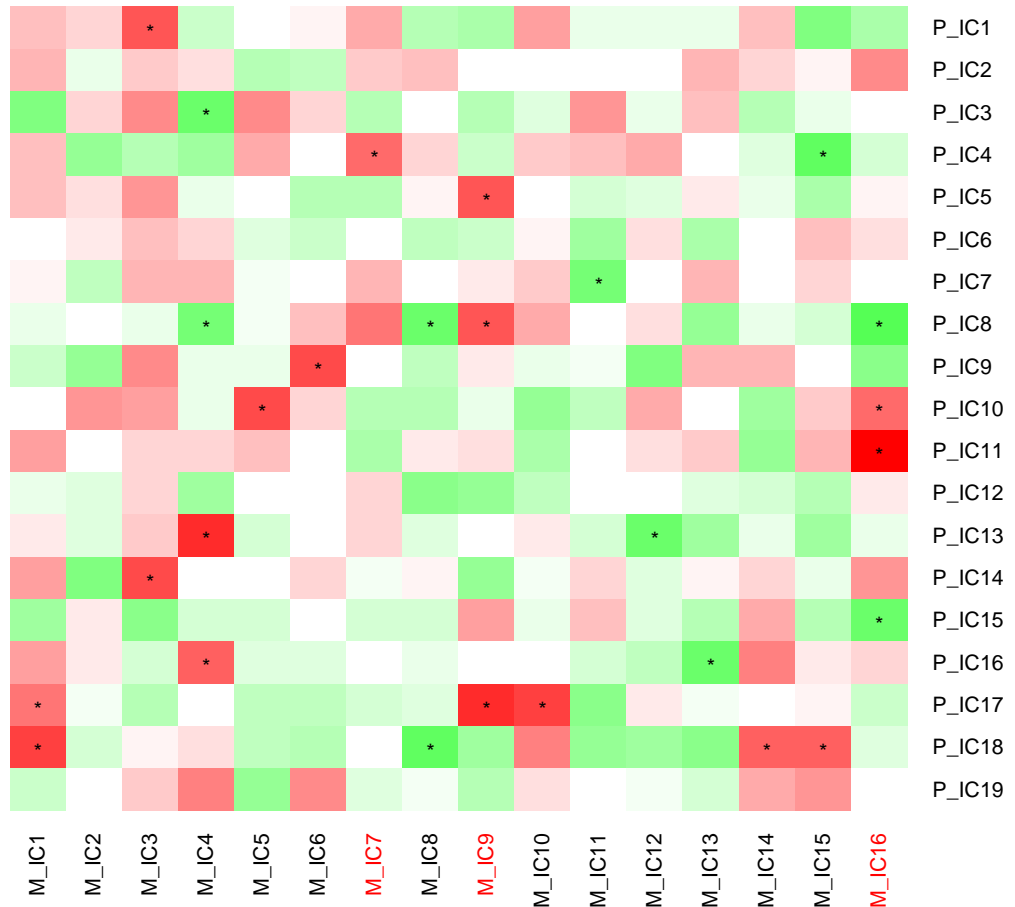

Color Key

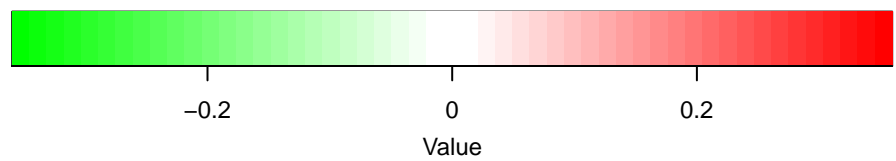

Supplement: Supplementary file 6 — Supplementary Figure 1 [file 41398_2022_1940_MOESM6_ESM.pdf]
